# A single blood test to rule out acute coronary syndrome

**DOI:** 10.1136/heartjnl-2017-312269

**Published:** 2017-10-27

**Authors:** Andrew R Chapman, Nicholas L Mills

**Affiliations:** BHF Centre for Cardiovascular Science, University of Edinburgh, Edinburgh, UK

**Keywords:** acute coronary syndromes, acute myocardial infarction

Strategies to improve the assessment of patients with suspected acute coronary syndrome continue to evolve, in recognition that fewer than 20% of those attending the Emergency Department with chest pain receive a diagnosis of myocardial infarction. Identifying patients without myocardial infarction at an earlier stage has the potential to reduce hospital admissions for serial cardiac biomarker testing, and facilitate appropriate investigation for alternative causes. However, such strategies are only helpful if it can be demonstrated that they do not compromise patient safety.

In 2016, the National Institute for Health and Care Excellence (NICE) updated their guidance on the evaluation of patients with suspected acute coronary syndrome. For the first time, they recommended clinicians consider ruling out myocardial infarction if a patient has very low concentrations of cardiac troponin at presentation when measured using a high-sensitivity assay.^1^ This guidance could lead to a significant reduction in the proportion of patients who require serial testing, and may tempt clinicians to consider upgrading their infrastructure to facilitate implementation. In the UK, two high-sensitivity assays are recommended by NICE for use in clinical practice, the Roche Elecsys high-sensitivity cardiac troponin T assay (hs-cTnT) and the Abbott ARCHITECT_STAT_ high-sensitivity cardiac troponin I assay (hs-cTnI). These assays measure different subtypes of cardiac troponin, and there are important differences in the normal reference range, diagnostic thresholds, levels of imprecision and in the lowest absolute concentrations which can be reliably detected, also known as the limit of detection (LoD) (table 1).

**Table 1:** 99th centile=the upper reference limit as determined in a healthy reference range population

	99th centile (Diagnostic threshold)	10% coefficient of variation*	Limit of detection
Roche Elecsys high-sensitivity cardiac troponin T^7^	14 ng/L	13 ng/L	5 ng/L
Abbott ARCHITECT_STAT_ high-sensitivity cardiac troponin I^8^	16 ng/L (females) 34 ng/L (males) 26 ng/L (single threshold)	4.7 ng/L	2 ng/L

Limit of detection, the lowest concentration which can be reliably distinguished from a sample with no troponin present.

*Lowest concentration where coefficient of variation is <10% (measure of dispersion of replicate sample results around the mean (SD/mean)).

NICE recommend clinicians apply the LoD as a threshold below which myocardial infarction can be safely ruled out at presentation. Such a strategy is only recommended for patients deemed to be at low risk of myocardial infarction ‘as indicated by a validated tool’. During their appraisal, NICE considered evidence from studies including both the Thrombolysis in Myocardial Infarction (TIMI) score and the Global Registry of Acute Coronary Events score. Both scores were derived and validated in patients with confirmed myocardial infarction to confer prognosis, but over time, these scores have been implemented for risk stratification in patients with suspected, not confirmed, myocardial infarction. Importantly, cardiac troponin concentrations are embedded in both risk scores. NICE ultimately recommend the TIMI score, which has been previously validated in patients with suspected acute coronary syndrome alongside a contemporary troponin assay and serial testing,^2^ but not with a high-sensitivity assay and the LoD at presentation alone.

Carlton *et al* provide the first validation of the NICE guidance in a pooled study of over 5000 patients, in five observational cohorts across two continents, with varying prevalence of major adverse cardiovascular events (4.8% to 15.6%).^3^ They found when an hs-cTnT of <5 ng/L (LoD) was applied alongside a TIMI score of 0 and a non-ischaemic ECG, the sensitivity and negative predictive value (NPV) were extremely high, at 99.5% (95% CI 98.1% to 99.9%) and 99.6% (95% CI 98.7% to 100%), respectively. They derived a meta-estimate for sensitivity of 98.7% (95% CI 96.5% to 99.6%), with low heterogeneity observed between cohorts (*I_2_* 15.3). For the hs-cTnI, using the LoD (<2 ng/L) and a TIMI score of 0 alongside a non-ischaemic ECG, the sensitivity was 98.9% (95%CI 97.4% to 99.6%) and NPV was 99.5% (95%CI 98.8% to 99.8%). The meta-estimate for sensitivity was similar (98.5%, 95% CI 95.4% to 99.5%) but the heterogeneity was high (*I_2_* 73.7). The reason for the observed heterogeneity is unclear, but may reflect differences in the assay used for diagnostic adjudication and testing between cohorts. These strategies would identify between 17.9% (95% CI 16.6% to 19.3%) and 21.0% (95% CI 19.9% to 22.2%) of patients as low risk, respectively. The authors evaluate several additional approaches not included in the recommendations of NICE, including the use of thresholds above the LoD in combination with the TIMI score, and their data suggest that higher thresholds (such as <7 ng/L on the hs-cTnT assay, or <5 ng/L on the hs-cTnI assay) could increase in the proportion identified as low risk without compromising sensitivity or NPV.

While NICE recommend use of the TIMI score, the true need for clinical risk scores in this setting is uncertain. A recent meta-analysis of 9269 patients found a normal ECG and an hs-cTnT result below the LoD provided excellent NPV (99.3%, 95% CI 97.3% to 99.8%) and sensitivity (98.7%, 95% CI 96.6% to 99.5%) for the diagnosis of myocardial infarction, without the need for additional risk scores.^4^ There were no deaths at 30 days in patients classified as low risk with the index test. This reflects our understanding that patients classically considered high risk (due to increasing age or cardiovascular risk factors such as diabetes, renal disease or prior ischaemic heart disease) have chronic elevation in high-sensitivity cardiac troponin concentrations (within the normal reference range) and are less likely to have low concentrations to support early discharge. Indeed, the European Society of Cardiology advocate use of the LoD at presentation in conjunction with the ECG, but do not recommend the addition of clinical risk scores.

However, there is undoubtedly an appetite for clinical risk scores in some settings, perhaps due to the additional perceived diagnostic confidence they provide. One of the most widely used risk scores, the HEART score, was developed and validated in a suspected acute coronary syndrome population. This score is based on clinical variables selected a priori (*H*istory, *E*CG, *A*ge, *R*isk factors, cardiac *T*roponin) with arbitrary weighting chosen on a pragmatic basis. A recent meta-analysis of 11 217 patients demonstrated this score had a sensitivity of just 96.7% (95% CI 94.0% to 98.2%), below the threshold of 99% which most emergency department physicians deem acceptable.^5^ Whether use of this score offers additional benefit over risk stratification with troponin alone is unclear. Comparative studies including risk stratification thresholds alone or in combination with risk scores are required to determine if improvements in safety can be obtained.

There are important limitations to the study findings of Carlton *et al.* It is notable that there was a significant proportion of missing data, with over 2000 patients excluded from the hs-cTnT cohort and 784 patients from the hs-cTnI cohort. This reflects the pragmatic basis on which the data were compiled for this study, but may have contributed to important differences in risk profile between cohorts. Patients in the hs-cTnT cohort were older, with more cardiovascular risk factors, and one may therefore anticipate higher baseline cardiac troponin concentrations and a reduced likelihood of a missed myocardial infarction compared with the hs-cTnI cohort.

So, is it time to adopt the NICE guidance? The evidence supporting implementation of strategies including low high-sensitivity cardiac troponin concentrations is strong, but some uncertainty remains. All studies on which the NICE recommendations are based were observational in nature (ie, no patients were discharged from hospital on the basis of a single troponin result), and the same applies to the vast majority of studies in this area. A multicentre randomised controlled trial of implementation of a rule-out strategy using a single hs-cTnI concentration (<5 ng/L) has completed recruitment in Scotland (NCT:03005158), and similarly, a randomised controlled trial of the LoD approach for the hs-cTnT is being planned (LoDED study, personal communication Dr Edd Carlton). Both trials will give complementary insight into the safety and efficacy of this approach.

For the clinician who plans to implement this strategy prior to the outcomes of these randomised controlled trials, there are some important considerations. Patients who present early after onset of symptoms are challenging to recruit and therefore under-represented in all observational cohort studies. It is therefore recommended that serial testing is performed in all who present early after onset of symptoms. Similarly, any patient with myocardial ischaemia on the ECG should not be considered for early rule out and should undergo serial troponin testing. Of utmost importance is an awareness of the assay in use at your institution, the normal reference range and the appropriate diagnostic and risk stratification thresholds which are not equivalent. Where low concentrations are reported, it is important to ensure appropriate standards for clinical reporting can be met and maintained under routine working conditions. As noted by NICE, implementation of the proposed early rule-out strategy should include clinical audit, with attention paid to the time taken to rule out the diagnosis and on the clinical outcomes of patients with suspected acute coronary syndrome.

Clinicians should be confident that newer approaches using low concentrations of cardiac troponin are a magnitude safer than prior strategies using the 99th centile alone at presentation and 3 hours as recommended in the European Society of Cardiology guidelines.^6^ Clinicians should be prepared to restructure the assessment of patients with suspected acute coronary syndrome in their institution to harness the potential of high-sensitivity cardiac troponin testing, and to improve the efficiency and safety of healthcare delivery.

## References

[1] National Institute for Health and Care Excellence. NICE guidance. Chest pain of recent onset: assessment and diagnosis (update). CG95 London. 2016 https://www.nice.org.uk/guidance/cg95. 32065740

[2] Than M, Cullen L, Aldous S, et al 2-Hour accelerated diagnostic protocol to assess patients with chest pain symptoms using contemporary troponins as the only biomarker: the ADAPT trial. J Am Coll Cardiol 2012;59:2091–8. 10.1016/j.jacc.2012.02.035 22578923

[3] Carlton EW, Pickering JW, Greenslade J, et al Assessment of the 2016 national institute for health and care excellence high-sensitivity troponin rule-out strategy. Heart 2018;104:665–72. 10.1136/heartjnl-2017-311983 28864718 PMC5890641

[4] Pickering JW, Than MP, Cullen L, et al Rapid rule-out of acute myocardial infarction with a single high-sensitivity cardiac troponin measurement below the limit of detection: a collaborative meta-analysis. Ann Intern Med 2017;166:715–24. 10.7326/M16-2562 28418520

[5] Van Den Berg P, Body R The HEART score for early rule out of acute coronary syndromes in the emergency department: a systematic review and meta-analysis. Eur Heart J Acute Cardiovasc Care 2017:204887261771078 [Epub ahead of print] 10.1177/2048872617710788 28534694

[6] Chapman AR, Anand A, Boeddinghaus J, et al Comparison of the efficacy and safety of early rule-out pathways for acute myocardial infarction. Circulation 2017;135:1586–96. 10.1161/CIRCULATIONAHA.116.025021 28034899 PMC5404406

[7] Giannitsis E, Kurz K, Hallermayer K, et al Analytical validation of a high-sensitivity cardiac troponin T assay. Clin Chem 2010;56:254–61. 10.1373/clinchem.2009.132654 19959623

[8] Shah AS, Griffiths M, Lee KK, et al High sensitivity cardiac troponin and the under-diagnosis of myocardial infarction in women: prospective cohort study. BMJ 2015;350:g7873 10.1136/bmj.g7873 25609052 PMC4301191

